# Knowledge and Beliefs about Herb/Supplement Consumption and Herb/Supplement–Drug Interactions among the General Population, including Healthcare Professionals and Pharmacists: A Systematic Review and Guidelines for a Smart Decision System

**DOI:** 10.3390/nu15102298

**Published:** 2023-05-13

**Authors:** Artemisa R. Dores, Miguel Peixoto, Maria Castro, Catarina Sá, Irene P. Carvalho, Andreia Martins, Eva Maia, Isabel Praça, António Marques

**Affiliations:** 1 Laboratório de Reabilitação Psicossocial, Centro de Investigação em Reabilitação (CIR), Escola Superior de Saúde, Instituto Politécnico do Porto (ESS-IPP), 4200-072 Porto, Portugalajmarques@ess.ipp.pt (A.M.); 2 Clinical Neurosciences and Mental Health Department, Faculty of Medicine, University of Porto, 4200-319 Porto, Portugal; irenec@med.up.pt; 3 CINTESIS@RISE, Faculty of Medicine, University of Porto, 4200-450 Porto, Portugal; 4 Research Group on Intelligent Engineering and Computing for Advanced Innovation and Development (GECAD), School of Engineering, Polytechnic of Porto (ISEP/IPP), 4249-015 Porto, Portugalegm@isep.ipp.pt (E.M.); icp@isep.ipp.pt (I.P.)

**Keywords:** knowledge, beliefs, perceptions: attitudes, herb/supplement–drug interaction (HDI), pharmacy

## Abstract

The increased consumption of a variety of herbs/supplements has been raising serious health concerns. Owing to an inadequate understanding of herb/supplement–drug interactions, the simultaneous consumption of these products may result in deleterious effects and, in extreme cases, even fatal outcomes. This systematic review is aimed at understanding the knowledge and beliefs about the consumption of herbs/supplements and herb/drug–supplement interactions (HDIs). The study follows the PRISMA guidelines. Four online databases (Web of Science; PubMed; Cochrane; and EBSCOhost) were searched, and a total of 44 studies were included, encompassing 16,929 participants. Herb and supplement consumption is explained mostly by the reported benefits across multiple conditions and ease of use. Regarding HDIs, most people take both herbs/supplements and prescription drugs simultaneously. Only a small percentage of participants have knowledge about their interaction effects, and many reported adverse interactions or side effects. Nevertheless, the main reason for stopping the prescribed drug intake is the perceived lack of its effect, and not due to interactions. Therefore, it is important to increase the knowledge about supplement use so that further strategies can be elaborated to better detect or be alert for whenever a potentially dangerous reaction and/or interaction may occur. This paper raises awareness regarding the need for developing a decision support system and ends with some considerations about the development of a technological solution capable of detecting HDIs and, thereby, aiding in the improvement of pharmacy services.

## 1. Introduction

Studies have shown the presence of a high prevalence of herbal medicine (HM) consumption, for example, among the population with hypertension [1], in the postpartum period [2], and among pregnant women [3,4], including dietary supplements (DS) [5,6,7,8,9]. HMs include herbs, herbal substances, and products of plants or a combination of plants that were used even before the discovery of conventional drugs [10]. DSs include ingredients such as vitamins, minerals, dietary herbs, amino acids, and enzymes [11]. The same phenomenon of increased consumption has been occurring as regards image- and performance-enhancing supplements or drugs (IPEDs), also known as lifestyle drugs, comprising various products, including anabolic steroids, sexual enhancers, growth hormones, and other drugs that can alter the functions of the body to enhance muscle growth, reduce body fat, and promote weight loss [12]. The consumption of this kind of product has increased worldwide, as shown in several papers (e.g., [12], namely during the COVID-19 lockdown). In a recent study involving seven countries, the participants reported the use of a wide range of drugs and medicines to boost their image and performance (28%), which was maintained during the lockdown, mostly in Hungary (56.6%), Japan (46.8%), and the UK (33.8%), or started taking new IPEDs (6.4%) [13].

The scientific community and clinical practitioners, namely the American College of Clinical Pharmacology [14], have raised serious health concerns due to potentially dangerous interactions that could occur between prescription medications and herbs/supplements [15,16,17]. Directly connected with the possible drug interactions are cytochrome P450 enzymes (CYP), which are essential for the metabolism of drugs [18,19]. The possible interactions arise when the ingested drugs have CYP activity, which can cause a variety of reactions, such as inhibiting or inducing CYP activity [18]. These enzymes are mostly found in the liver and, depending on their pathway, can have a different designation (i.e., CYP1, CYP2) [18]. Studies show that certain herbal products have an effect on CYP pathways, such as inhibiting their activity [20], which can lead to potential Herb/supplement–Drug Interactions (HDIs) when administering drugs that are metabolized by the same enzyme [20,21,22]. Studies also show that the P-glycoprotein (P-gp), an ATB-binding cassette transporter responsible for taking toxins and xenobiotics out of cells [23], is also affected by various Natural Products (NP) [20,24], which can result in its inhibition [25]. Herbs that affect P-gp also show effects on CYP, further validating the importance of these two compounds on HDIs [25].

Because of the effects of HDIs, researchers have increasingly been investigating several products that can result in interactions, and their effects. One example is Licorice, an herb that is thoroughly investigated for its pharmacological benefits, and that is consumed in a variety of products such as sweets, cakes, beverages, and teas. However, despite its recorded benefits, Licorice also inhibits CYPs. Given the wide variety of species of Licorice, they can affect different CYPs, with different degrees of severity [26]. Similar to Licorice, Grapefruit also alters CYPs, namely in the intestinal wall [27], due to the high dose of 6′,7′-dihidroxybergamottin (DHB) that they contain [28]. Grapefruit consumption has also been shown to increase the levels of simvastatin and lovastatin, and, to a lesser degree, the levels of atorvastatin [28]. St John’s wort also potentiates HDIs, by altering the pharmacokinetics of various drugs, through the activation of PXR [29].

The Accreditation Council for Pharmacy Education in the US has been recommending formal training on herbal and on complementary and alternative medicine (CAM). However, this training is not mandatory in the formal academic-based curricula of Graduate Degree Programs for Pharmacy and Pharmaceutical Sciences [14]. Most community pharmacists have a positive attitude regarding the use of herbs/supplements and show good practices toward these products, recognizing the possible adverse effects of HDIs and related contraindications [30]. Pharmacists are in the unique position of being in contact with the public due to their proximity to the communities, and they have the ability to follow-up with the user for longer periods when compared to many other healthcare professionals [31]. They can thus educate patients about the use, efficacy, side effects, and potential interactions of these products with prescription medications [32,33]. However, given that the acquisition of herbs/supplements is not restricted to pharmacies, the major concerns about the effects of these products should be directed at the general population, among whom knowledge about the potential risks of herb/supplement consumption is limited [34]. This lack of information is not limited to herb/supplement consumption but also includes the HDIs. This interaction is defined as the pharmacological response to the administration of, or co-exposure to, a drug with another substance that modifies the patient’s response to that drug [35], which can result in serious health problems [17,36]. For this reason, the general population’s lack of knowledge regarding this interaction effect has been of concern to public health authorities [37]. In other words, the lack of knowledge regarding supplements, together with concerns about changes in body image, can lead to harmful behaviors that could promote the intake of supplements that guarantee immediate results in terms of body transformation [13]. The possible interactions of these substances with other prescription drugs and the associated health risks raise the need for new types of interventions by healthcare professionals, namely pharmacists [38,39]. 

In addition to this general lack of knowledge regarding HDIs and self-medication behaviors in the general population, the fact that these herb/supplements are referred to as “natural”, and do not require a medical prescription, creates the misconception that their effects are harmless, and partly explains the increase in their consumption over time [34]. Although there is limited evidence about the use of these supplements in the prevention or treatment of diseases [40], the marketing of these herbs/supplements has been increasing, as previously mentioned [15,41,42].

The commercialization of these products occurs rapidly, making it difficult for healthcare professionals and pharmacists to respond in a scientifically supported way to these advances. Furthermore, “the lack of strict government regulation of supplements, accompanied by consumers’ lack of knowledge, often results in consumers relying on common lay (naïve) beliefs and theories when making supplement-based decisions” [43]. 

According to the theory of social representations [44], a social representation is a system of values, ideas, and practices that are acquired through communication and interactions among group members and between members and institutions, such as the media. This communication is carried out through two processes, namely, anchoring and objectification. Through social representations, individuals can acquire collective cognitions that fit within a certain group. In the case of CAMs, the representations that society has about these types of products, and about health and illness, can be integrated within the person’s viewpoint [45]. Thus, the knowledge, attitudes, and beliefs of the individual are influenced by the community, even if the person has a certain degree of autonomy. These representations are conveyed through communication, namely, through the processes of anchoring and objectification [45]. Anchoring refers to the process by which beliefs about supplements and herbal medicines are anchored on something that was already known to facilitate the understanding of the new phenomenon. Although anchoring facilitates an increased understanding of the new phenomenon through the already known information, it can also create erroneous beliefs about the new phenomenon. For example, a study showed that about 69% of people think that cancer can be controlled with naturally available herbs [10]. Despite the potential importance of these products, their use might be dangerous. Concerning objectification, its aim is also to facilitate communication, but through visual and linguistic tools that serve to describe and make the belief psychologically tangible. For instance, the concepts of herbs and supplements are associated with medicine, and with natural and nutritional terms. Therefore, anchoring and objectification will contribute to the development of social representations about herbs/supplements in the population, thus integrating them in individuals’ behaviors, lifestyles, and routines. If the representations are too divergent from the reality of the herb/supplements’ effects from the reality of their interactions with prescription medications, then, the interventions for behavioral changes in the population, regarding the intake of these substances, may be inappropriate or ineffective. For example, if herbs/supplements are anchored on natural substances, the prevention of interaction consequences will be ignored. To get to social representations, it is thus important to explore the beliefs and attitudes of the population about supplements and other herbal medicines. 

Despite some studies exploring herbs/supplements and medications [46,47,48,49], this information is not reviewed and organized in a systematic manner that allows the medical community and other governmental entities to access and interpret the evidence clearly, and to objectively formulate recommendations to address this problem. 

Therefore, the aim of this study was to perform a systematic review on the beliefs, attitudes, knowledge, or perceptions about the consumption of herbs/supplements and HDIs, and their impact on the intake of these products, among the general population, including healthcare professionals and pharmacists. 

The following questions guided this review:

Question 1: What is the knowledge, beliefs, attitudes, or perceptions regarding the consumption of herbs/supplements?

Question 2: What is the knowledge, beliefs, attitudes, or perceptions regarding interactions between herbs/supplements and other drugs?

Question 3: What is the knowledge, beliefs, attitudes, or perceptions regarding the effects and risks of herbs/supplements?

Answering these questions will contribute to a better understanding about the needs of the general population and of the medical community on this topic, given that population perspectives have been shown to be a predictor of the intention to adhere to a service [50]. Therefore, the understanding of the perspectives on HDIs will increase the comprehension of the necessary factors to consider when formulating support strategies in this area, effectively responding to real needs. Additionally, as shown by Wilson et al. [51], an effective expansion of the services provided by a pharmacy is correlated with an approach that seeks to know and to supply services that are of value to the patient. Moreover, this type of approach shows a correlation, mediated by the efficiency in service expansion, with pharmacists’ performance. Another aspect to take into consideration in the implementation of support strategies is adherence. The implementation should integrate the capacity to respond to the factors that govern adherence to treatment, which include the understanding of beliefs, values, attitudes, and motivation/capability to do so. The consideration of these factors will allow the development of support strategies that promote a higher degree of adherence and that take into consideration the needs of the people who look for them [52].

Based on the results obtained in this review, some considerations about the development of support strategies, in the form of a Clinical Decision Support System (CDSS), are presented. These types of systems are of paramount significance for healthcare professionals, who rely predominantly on biomedical scientific literature as their primary source of information. These systems can supplement and augment the knowledge of healthcare professionals, assisting them in making informed decisions when providing care to their patients. Therefore, investing in the development and implementation of such systems should be considered a top priority of healthcare organizations for informed decision-making and improvement in patient care. Moreover, the available literature on herbs/supplements and HDIs is constantly updated, making it difficult for experts to thoroughly review it all. Therefore, the existence of dependable resources that can keep healthcare professionals up to date on possible harmful interactions is crucial [53]. 

In the past, owing to a variety of factors, health-related difficulties caused by HDIs often went unnoticed by physicians. One such factor is the insufficient knowledge among healthcare professionals concerning herbs/supplements and their potential for drug interactions. Additionally, many patients did not perceive it as necessary to disclose their herb/supplement consumption to their healthcare professionals, who themselves seldom inquired about this aspect of their patient’s health history [54].

Since most of these herb/supplement products do not necessitate a prescription and are readily available in pharmacies or para-pharmacies, the pharmacy community, which frequently serves as people’s initial point of contact with the healthcare system, is a well-suited context to identify potential consumers early on, educate them on supplement usage, and subsequently reduce the risk of harmful interactions while promoting public health. The idea of developing a CDSS emerged in the face of this predicament. This system will serve as a critical tool to assist pharmacists in converting large volumes of clinical data into useful knowledge that can be readily available for consultation and action. It will help increase awareness about possible interactions, aid in making informed treatment decisions, and mitigate any potential adverse drug reactions (ADRs) [55].

## 2. Materials and Methods

The recommendations of PRISMA-Preferred Systematic Review and Meta-analysis [56] were followed to guide the general stages and protocols of this review.

### 2.1. Search Strategy

A systematic literature search was performed to identify studies about beliefs, attitudes, knowledge, or perceptions about herb/supplement consumption and HDIs. Studies were identified through the search of multiple literature databases in PubMed, Cochrane, EBSCOhost, and Web of Science.

This database search was complemented by additional hand searching of referenced studies in other articles to prevent publication and source selection bias. The keywords and search string were: “knowledg* OR belie* OR myth* OR perception* OR Attitude* OR Cognition*” (TI Title); “vitamin supplement*” OR “dietary supplement*” OR “drug supplement*” OR “medication supplement*” OR “supplement consumption” OR phytodrug* OR herb* OR plant* OR vitamin OR “Image and Performance Enhancing Drug” OR IPED OR “Performance and image enhancing drug*” OR “PIED” (in abstracts); complications OR interactions (AB Abstract); pharmacolog* OR med* OR drug OR “nutrient-drug” (AB Abstracts). The search was limited to English, Portuguese, and Spanish.

### 2.2. Study Selection

Inclusion criteria consisted of the consumption of herbs/supplements with medication; studies in scientific, academic, and peer-reviewed journals; articles between the years 2012 and 2022; and studies in English, Portuguese, and Spanish. Exclusion criteria were articles that did not mention the variables in the research question and studies that did not investigate beliefs, attitudes, or knowledge about supplements. Duplicate articles were eliminated, as well as articles published before 2012. 

Two independent reviewers conducted the selection of the studies (M.C. and C.S.D), according to the Cochrane Collaboration’s recommendations [57]. The Rayyan Intelligent Systematic Review tool [58] was used to ensure the blind application of the inclusion/exclusion criteria. An inter-rater agreement value of 0.853 was obtained. The decisions by the two independent reviewers were compared, and disagreements were resolved through discussion. 

## 3. Results

**Figure 1:**
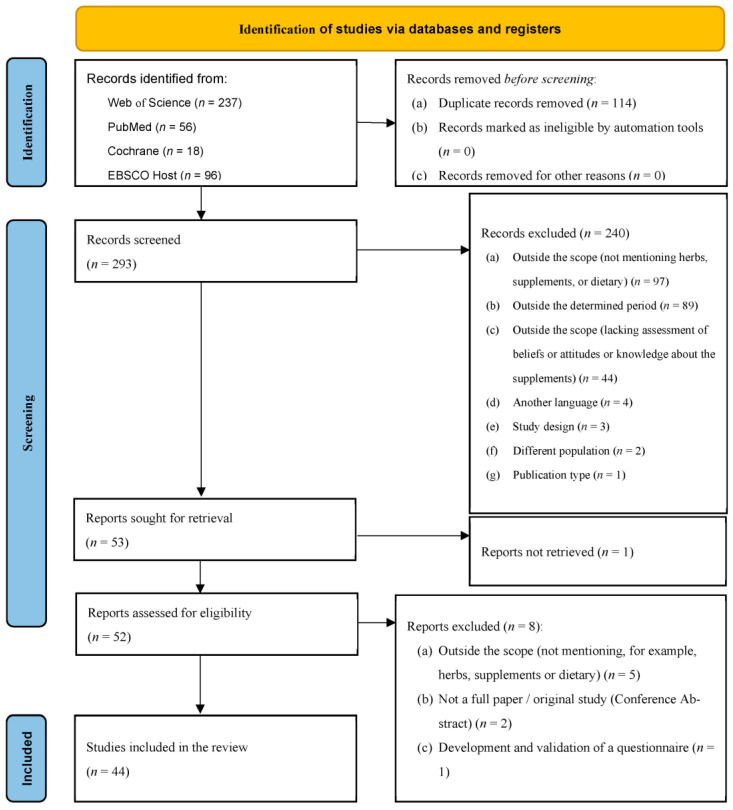
PRISMA flow diagram of the literature search.

A total of 407 articles were identified in the selected databases (see Figure 1), namely, Web of Science (*n* = 237), PubMed (*n* = 56), Cochrane (*n* = 18), and EBSCOhost (*n* = 96). After the exclusion of duplicates, the titles and abstracts of 293 articles were analyzed. Of these, 240 articles were excluded due to the following reasons: (a) Not mentioning the variables in the research question (e.g., herbs, supplements, or dietary) (*n* = 97); (b) Outside the determined period (*n* = 89); (c) Outside the scope of the review (lacking assessment of beliefs, attitudes or knowledge about the herbs/supplements (*n* = 44); (d) Other than the intended language (*n* = 4); (e) Non-empirical studies (*n* = 3); (f) Different population (i.e., animals) (*n* = 2); and (g) Publication type (i.e., dissertation, thesis, commentary articles) (*n* = 1). A total of 53 articles were sought for retrieval, with one not being available. A total of 52 full-text articles were read in full and some were excluded for being outside the scope of the review (not mentioning herbs, supplements or dietary) (*n* = 5). Others were excluded for not being considered as a full article/original study (conference abstract) (*n* = 3), and another was excluded for consisting of the development and validation of a questionnaire (*n* = 1). Overall, 44 papers met the eligibility criteria for inclusion in the systematic review. The two independent reviewers obtained a Cohen coefficient of 0.853, indicating almost a perfect agreement in the selection process [59].

### 3.1. Study Characteristics

The design of the studies was described based on the types organized in [60]. Most studies used a correlational/comparative design. Specifically, 37 followed a cross-sectional design [2,7,10,30,61,62,63,64,65,66,67,68,69,70,71,72,73,74,75,76,77,78,79,80,81,82,83,84,85,86,87,88,89,90,91,92,93] (Table 1) and two followed experimental designs [43,94] (Table 2). Three studies used qualitative methodology [95,96,97] and two used mixed-method (qualitative and quantitative) research designs [98,99] (Table 1). The studies’ results concerning knowledge, beliefs, perceptions or attitudes about supplements, interactions, and risks/adverse effects are shown in Supplementary Material, Table S1 (correlational/comparative studies) and Table S2 (experimental studies). 

The studies included 16929 participants (*M* = 384.68 participants, *SD* = 578.98, Min. = 12, Max. = 2830). Regarding gender distribution, 7683 were female participants (45.39%), 8410 were male participants (49.68%), and the gender of 856 participants was not reported (5.06%). Several studies [10,61,67,68,69,74,78,83,98] did not include complete or any information on the gender of the participants. Some studies [10,61,63,74] did not include information on the age of the participants. To the authors’ knowledge, the age of the participants ranged from 14 to 88 years. 

**Figure 2:**
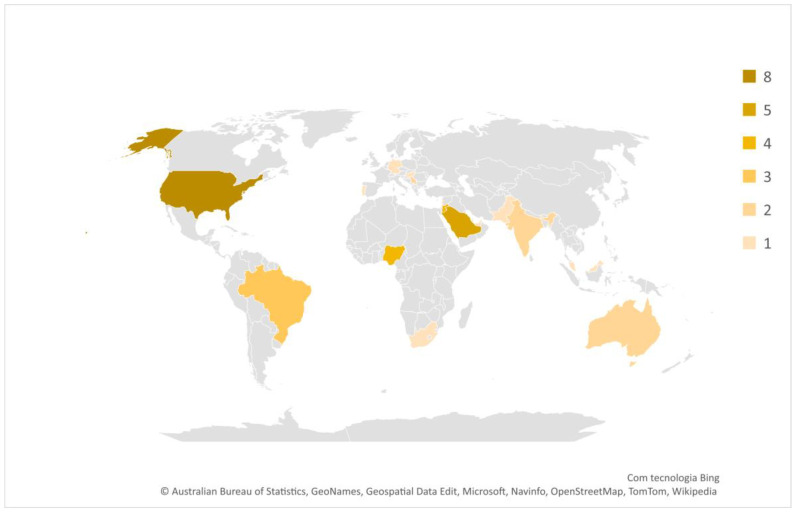
Countries where the studies were conducted.

Regarding the countries where the studies were conducted, Figure 2 highlights their geographical distribution. The data presented shows that the number of studies per country is the following: USA (*n* = 8); Saudi Arabia (*n* = 5); Jordan (*n* = 4); Nigeria (*n* = 4); Lebanon (*n* = 3); Brazil (*n* = 3); India (*n* = 2); Serbia (*n* = 2); Australia (*n* = 2); Hungary (*n* = 1); Pakistan (*n* = 1); South Africa (*n* = 1); Portugal (*n* = 1); Malaysia (*n* = 1); England (*n* = 1); Germany (*n* = 1); United Arabs Emirates (*n* = 1); Palestine (*n* = 1); and across several countries (*n* = 2).

### 3.2. Instruments of Data Collection

Regarding data collection procedures, most of the data were collected through questionnaires/surveys [7,10,30,61,62,63,64,65,66,67,68,69,70,71,72,73,74,75,76,77,78,79,80,81,82,83,84,85,86,87,88,89,90,91,92,93,98]. In other studies, data were collected through interviews [2,61,96,99] and three of these studies used focus groups [92,95,97].

### 3.3. Procedures for Data Collection

For NP and/or NS and/or DS, for HM, and HDIs, domains such as knowledge, perception, beliefs, and attitudes of the general population, including healthcare professionals and pharmacists, were assessed. Knowledge, beliefs, attitudes, or perceptions about the use of NP, NS, DS, and HM were found in 41 studies [2,7,10,30,43,62,63,64,65,66,67,68,69,70,71,72,73,74,95,98,99]. The interactions were found in 24 studies [7,30,61,62,63,67,68,69,72,73,75,78,79,81,82,83,84,85,86,87,90,93,94,97].

Regarding the knowledge, beliefs, attitudes or perceptions about the risks of supplement use, or interactions associated with the use of other medicines, 12 studies assessed how people perceive the risks of both use and interactions [7,63,69,73,77,82,84,85,87,88,94,99].

In terms of the participants, 13 studies included pharmacists [30,61,69,70,73,74,75,80,82,86,89,90,94], 12 studies included the general population [10,43,62,68,72,76,84,85,88,92,95,98], 11 studies included a variety of healthcare workers [63,66,67,71,75,77,78,79,81,96,99], and 9 studies included patients with various conditions [2,7,64,65,83,87,91,93,97].

### 3.4. Main Results

#### 3.4.1. Herb/Supplement Consumption

Considering the knowledge, beliefs, attitudes, or perceptions toward herb/supplement consumption (Question 1), the results converged as regards the recognition of benefits associated with the intake of these products. For example, HMs were considered to be useful [61,69,89] and safe [30,82] by pharmacists and by the general population (respectively, useful [62,84,85] and safe [62,68,88]). In addition, they were considered to be easy to use by the general population [62,88]. Other healthcare professionals also considered these products to be useful [77] and easy to use [81], and patients considered them to be safe [7,64,65,87,93] and easy to use [65].

Other studies showed that the general population [10,62,72,76,84,85,88,92,95,98], patients [2,7,64,91,97], and pharmacists [30,70,86] reported that herbs/supplements prevent and/or cure diseases, including diabetes [30,64,76,88], and alleviate other health problems, such as constipation and acne [64], or cancer [10,30,91]. In other studies, the general population used these products to help combat COVID-19, to prevent contagion, and to cure it, along with other medications [92,98]. Pharmacists [30,88] and the general population [95] used these substances for the home treatment of hypertension and related symptoms. Patients [2,7,65] and the general population [72] used them to increase disease control. Some reported that supplements were important for maintaining a healthy life, namely the general population [72,84,88,92], patients [64,91], and healthcare workers [78]. 

In their study, Ceremuga et al. (2020) [7] reported a panoply of understandings about the functions of herbs/supplements among patients, some of which were described above. For example, in patients’ views, these products were beneficial for low-level deficiencies, fertility, overall health, brain health, breast feeding, energy, diabetes and blood glucose regulation, depression, sleep, relaxation, joint pain and arthritis, increased metabolism, heart health, eye health, post-workout muscle-strength recovery, gastrointestinal health, menopause and hot flashes, good skin–hair–nails, prevention of urinary tract infections, increased immunity, prostate health, sexual enhancement, weight loss, aging, decreased cholesterol levels, migraines, anti-inflammatory action, memory, post-bariatric surgery, lowered blood pressure, post-traumatic stress disorder, chemotherapy, liver, nausea, and increased circulation and blood flow. Studies have also reported the use of herbs/supplements without technical advice or supervision, by both the general population [62,95] and patients [64,65]. In other studies, herbs/supplements were used by patients in view of the limited benefits obtained from conventional medicines [65]. In some studies, patients reported having a more natural treatment with the use of herbs/supplements, and others used these products as a complementary measure to treatment [97]. For pharmacists, herbs/supplements were used for maternal health purposes, such as to reduce colic and treat hemorrhoids [30]. For patients, they were used for increasing lactation and for weight control [2]. For healthcare workers [66,99], pharmacists [73], and the general population [95], HM was considered to be the most frequently recommended treatment [66,73,95,99]. Some participants, such as pharmacists [69,86] and healthcare professionals [71], were not sure if they had used HM [69,71,86], or if they have any knowledge on herbs/supplements (some healthcare professionals) [71,79]. In other studies, the general population [68], pharmacists [82], and patients [83] had no knowledge about herbs/supplements at all, or little specific training on these products [10,78,96], including healthcare professionals [63,67,70,71,78,96], nurses [70], pharmacists [69,70,74], and the general population [10,30,64]. 

Additionally, some studies examined participants’ confidence in communicating knowledge about herbs/supplements, among both the general public [43] and pharmacists [89,90], in particular, about some of the benefits that certain types of supplements have on people’s lives. For example, in community pharmacists’ perspectives, Echinacea is used to boost immunity; St John’s wort is commonly used for mild to moderate depression; Arnica is used for minor skin irritations and bruising; Ginger is used for motion sickness, nausea and pregnancy-associated vomiting; Ginkgo delays dementia; and Chamomile is indicated for inflammation, anxiety, and insomnia [90]. 

Some studies reported that the general population [43,88], pharmacists [70], and healthcare professionals [96] were aware of the laws and regulations controlling the use of herbs/supplements, in contrast with other studies in which healthcare professionals had no such knowledge [71]. 

#### 3.4.2. Interaction between Herbs/Supplements and Prescription Drugs

Considering the knowledge, beliefs, attitudes, or perceptions regarding interactions between herbs/supplements and prescription drugs (Question 2), in some studies, a large proportion of participants used herbs/supplements and medications simultaneously [65,83,87]. In other studies, patients [65], pharmacists [74,75], and the general population [68,72] believed that herbs/supplements can interact with prescription drugs and with other herbs/supplements [93], and that some groups (in one study, the elderly and children) were more likely to experience interactions when compared to other groups [72].

Some studies have shown that pharmacists [89,90] and other healthcare professionals [77] are aware of the risks of possible interactions. In some studies, pharmacists reported the possibility of Ginkgo increasing the risk of bleeding when combined with Warfarin, and the cautionary use of Valerian if patients are using benzodiazepines [90]. In other studies, pharmacists reported that the risks associated with HDIs with medications included, for example, increased bleeding, heart or blood pressure problems, and altered mental status [30]. Those studies that provided more detailed data showed some of the better-known interactions by healthcare professionals, namely, doxycycline and levofloxacin antibiotics with magnesium and iron [79]. 

In contrast to the above studies, some studies have also reported a lack of knowledge, in the general population, about the synergistic effects of the interactions between herbs/supplements and prescription medications, and about the interactions of herbs/supplements with other herbs/supplements [68]. Some patients stopped their medications when using herbs/supplements only because they felt that the medications had not improved their health [65]. In other studies, pharmacists [61,80] and healthcare professionals [77,78,79] believed that interactions between herbs/supplements and conventional medicines can cause adverse reactions due to the lack of a high rate of recognition of the interactions [30,68,70,78,79,81,84,86,87]. Despite being open to their use, healthcare professionals reinforce the need for more research about the safety and effectiveness of herb/supplements, as well as more regulation of herbal practitioners, and more regulation on product quality and possible interactions [96].

#### 3.4.3. Effects and Risks of Herb/Supplement Use

Regarding the knowledge, beliefs, attitudes, or perceptions about the effects and risks of herbs/supplements (Question 3), the studies reported conflicting results among the different target groups. For example, patients considered herbs/supplements to be completely safe and as having no side effects [65], or showed no knowledge about such effects [87]. In contrast, pharmacists were aware of the risks associated with taking supplements [30,90]. As an example, pharmacists [89,90] and other healthcare professionals [77] observed that gastrointestinal consequences, such as diarrhea and obstructive constipation, were the most commonly experienced side effects in CAM therapies with the general population [88]. Pharmacists also showed awareness about the possibility of St John’s wort increasing the level of digoxin (a highly carcinogenic organophosphate) in the blood, and reported that Maca root should be avoided in patients with goiter, Ginseng can increase blood pressure [90], and Green tea was associated with insomnia [30,89]. The supplements classified as most dangerous by healthcare professionals were St John’s wort and Ginkgo, whereas creatine and vitamin C were perceived as safer [77]. In other studies, pharmacists have reported that Garcinia, Green tea, and Chromium are more dangerous, due to their potential risks [89]. In one study, some consumers perceived that certain types of supplements have more serious side effects than others [43]. In another study, healthcare professionals reported that the incorrect use of herbs/supplements can cause health risks [99], specifically, the risk of overuse of medicinal plants and herbal medicines, and the risk of misuse according to the type of plants that can be cooked and those that cannot [99]. Finally, about perceived risks, studies have reported that a lack, or a low level, of knowledge about herbs/supplements and about HDIs may result in inappropriate advice by the pharmacists [70,73,74,80].

## 4. Discussion

The main aim of this review was to explore the knowledge, beliefs, attitudes, or perceptions held by different parties (i.e., general population, including healthcare professionals and pharmacists) regarding the consumption of herbs/supplements, specifically, to determine the beliefs or knowledge about (1) the consumption of herbs/supplements, (2) interactions between herbs/supplements and medications, and (3) the effects and risks associated with the use of herbs/supplements. The review followed the PRISMA guidelines [56,100] and the recommendations of the guidelines for reporting systematic reviews [56]. 

With respect to the methodology of the reviewed studies, most followed a correlational/comparative design (*n* = 37), others followed experimental designs (*n* = 2), and the remaining studies were divided into using qualitative methodology (*n* = 3) and mixed methods (*n* = 2). As for the characteristics of the participants, the majority were male participants (*n* = 8410), with relatively fewer female participants (*n* = 7683). The number of participants per study averaged 384.68, and most were in an institutional or hospital setting.

Concerning the first question in this review, “What is the knowledge, beliefs, attitudes, or perceptions regarding the consumption of herbs/supplements among the general population, including healthcare professionals?”, the literature is consensual regarding the value of the supplements. Most of the studies reported that individuals believe that supplements prevent, control, and cure diseases. These beneficial effects are extended to various medical conditions, from constipation to cancer. In addition to physical health, the benefits are also extended to mental health, for example, depression. Considering the theory of social representations [44] described in the introduction, the evidence in the studies prevents the exploration of how the anchoring process was developed. It is unknown which terms would be anchored to facilitate the communication of the values, ideas, and practices associated with the concepts of herb/supplements among group members, institutions, and the media. Similarly, the objectification process is also unclear because data about some visual or/and linguistic tools that have served to describe and make the belief psychologically tangible were not found in the studies. The concepts of herbs/supplements may be associated with terms such as health, medicine, and cure, but this is speculative because there was no systematic search for theoretically driven knowledge in the studies. 

In relation to the second research question, “What is the knowledge, beliefs, attitudes, or perceptions regarding interactions between herb/supplement and drug consumption?”, most studies have shown that a large part of the population uses herbs/supplements simultaneously with prescription drugs. Most lack knowledge about HDIs, as well as interactions between different supplements. In addition, studies have reported that some people have stopped taking prescription drugs, not because they knew about the interactions, but because they did not have an effective response when they took their prescription drugs. Other studies have reported that people have experienced adverse reactions from the interactions between herbs/supplements and medications, or between different herbs/supplements. Specific risks reported from HDIs included increased bleeding, cardiac or blood pressure problems, and altered mental status [90]. Other studies even worked on the specific interactions between medicines and/or antibiotics with the use of some better-known herbs/supplements to prevent the repercussions of these interactions [77,79,89]. However, these studies are very specific and do not cover all the possible herbs/supplements or drug interactions that may occur and be used. Therefore, there is a gap between what is known and what remains to be known. 

Many studies call for the creation of training and education programs for professionals that prescribe both herbs/supplements and medicines, as well as for the sellers of these products. Some studies also recommend education about these products directed at the general population. The studies show that much of the knowledge about herbs/supplements in the general population comes from family, friends, the Internet, and the media. In comparison, healthcare professionals’ source of knowledge is mostly pharmacological education, product representatives, and information included in the products’ packages. These products are easily accessible, without government regulation to control their sales [43]. In addition to believing that these products are safe because they are natural, people also use them due to the belief that they are cheaper and easier to use than is conventional medicine. This review has shown that the rate of awareness about herbs/supplements and their interactions is very low. 

Concerning the third research question, “What is the knowledge, beliefs, attitudes, or perceptions regarding the effects and the risks of herbs/supplements?”, many studies have explored the risks or adverse effects of herb/supplement use. However, whereas some studies reported that HM was safe [7,93], others reported that no knowledge existed about its effects [65,87], and others showed that awareness existed about the risks associated with herb/supplement intake [7,90]. These studies identified side effects such as gastrointestinal effects (e.g., diarrhea and obstructive constipation), insomnia, and risk of bleeding, associated with the use of herbs/supplements [7,77,88,89,90].

The existing empirical consensus reinforces the need to inform and educate both the users of herbs/supplements and the professionals who prescribe and sell them. Informed supervision by healthcare professionals could help to prevent the potential side effects of herbs/supplements and of their interactions. Such an approach emphasizes the importance of these professionals having means, namely technologies, that allow them to provide informed supervision on herb/supplement intake and possible interactions, to increase the safety of the users.

These results highlight the need for the development of intelligent decision systems (CDSS) that can aggregate knowledge about the different interactions between herbs/supplements and medications, so that this information is available for healthcare experts to provide to the users. Such systems are even more important for HDIs because the latter are less studied than are Drug–Drug Interactions (DDIs). As the results show, it is crucial to raise awareness among consumers, clinicians, pharmaceutical industries, and health authorities regarding the risks associated with combining CAM with conventional drugs, in the same way that is already practiced with drug combinations [101,102].

Since most of these products can be obtained without a prescription from pharmacies or para-pharmacies, pharmacists are well positioned to identify potential interactions, provide guidance to consumers about the proper use of herbs and supplements, and ultimately reduce the risk of HDIs, promoting overall public health. For this goal to be accomplished, it is essential to have dependable resources that enable healthcare professionals to remain current and acquire pertinent information efficiently and promptly. They can then educate patients immediately following their purchase at the pharmacies. Therefore, pharmacists, who are people’s first line of contact with the healthcare system, can have a unique role to play in preventing DDIs, as well as HDIs [103]. 

The increase in the volume of biomedical literature regarding HDIs has prompted the scientific community to create a standardized methodology, or system, that utilizes Artificial Intelligence (AI) techniques to identify HDIs within textual data. Trinh et al. (2018) [104] suggested a clustering approach based on semantic relationships to identify possible HDIs in the biomedical literature. This involved, first, identifying the most pertinent herbal and drug entities and subsequently applying an unsupervised extraction method to cluster all potentially related pairs of entities. Essentially, this method groups entity pairs based on their relationship type, with different clusters representing distinct types of relationships. 

To mitigate other issues that arise when searching for articles related to HDIs, Lin et al. (2016) [105] have developed an automated PubMed-based article retrieval system for HDIs. This system eliminates the need for users to write a PubMed query by accepting medication and herb names as input and returning only the relevant articles.

Regarding dietary supplements, there is no complete information about how they interact with drugs, and its consumption has been increasing. To tackle this problem, Wang et al. (2019) [106] developed an application called SUPP.AI. The application uses a pre-trained language model called RoBERTa to extract information from scientific literature on HDIs. To train the model, the researchers used data from a related task of identifying DDIs assisting them in refining the language model and identifying HDIs more accurately. As a result, the SUPP.AI application allows users to search for evidence of these interactions and aims to close the information gap on dietary supplements, providing the most recent data on HDIs for healthcare professionals, scientists, and consumers to access easily. This research provides proof that some AI techniques, which have been suggested for managing DDIs, are also potentially effective when used in the context of HDIs.

Pharmacists frequently use CDSS to identify and prevent adverse drug reactions (ADRs). Medical Expert Systems (ESs) is a branch of AI that aims to imitate human thinking using computer technology. Its goal is to provide clinical decision support to healthcare professionals, patients, and other individuals at specific times to enhance the quality and safety of healthcare. This system is capable of collecting knowledge from an expert and then convert it into a knowledge base. This knowledge base encodes the expert’s knowledge into a set of if–then rules, similar to the way humans express their knowledge. Moreover, these systems exhibit higher precision and accuracy, when compared to humans, because they do not experience limitations such as forgetfulness, fatigue, or lack of expertise [107].

To tackle the vast amount of information related to DDIs, certain studies have employed this knowledge-based approach. For example, Kinney (1986) [108] developed an expert system using a microcomputer to assess its effectiveness in predicting DDIs in hospitalized patients. The system was able to predict 27 interactions, of which 10 did occur and were the cause for hospital admissions. In this way, clinicians were able to easily adjust the offending medications once they were made aware of the interactions. In a different study, Roach et al. (1985) [109] developed an expert system to structure and encode pharmacological information into rules and tables for systematic retrieval. This approach made the information easily accessible through natural language and a menu-driven interface. Thus, clinicians were able to use this system to understand the possible consequences of combining two drugs, why it occurs, and how harmful interactions can be alleviated. Additionally, the system provides information on related drugs that may also be involved in similar interactions. Still to answer the challenge regarding DDIs, Mahdi et al. (2018) [110] introduced a consultation tool to aid in prescribing medications and minimize the risk of potential drug interactions. The expert system incorporates the Cat Swarm Optimization Algorithm, allowing it to deduce conclusions from complex interactions and identify all possible interactions and their negative effects based on the drug input. Additionally, the system can suggest alternative medicines based on the patient’s medical history and conditions.

Despite all the described efforts, as far as we know, there has been no research that has implemented expert systems in the field of HDIs. Given the abundance of information available on potential HDIs, it has become clear that it is crucial to create such a system that enables healthcare professionals to stay informed. To address this need, and triggered by the results obtained in this research, an original and hybrid CDSS will be proposed in a future study to identify HDIs by applying AI techniques to identify new possible interactions [55]. In addition to the standard rule engine, the system will leverage the capabilities of ML models to enhance its performance. By using this system, pharmacists can enhance their awareness of HDIs and reduce the risk of ADRs. It is worth highlighting that the newly developed system will be entirely scalable to other interactions, such as DDIs and HDIs. This versatility has the potential to enhance the functionality of pharmacy systems.

Regarding the limitations of this study and future directions, the scientific evidence on beliefs is different across the different studies, making it difficult to integrate the evidence into a coherent and comprehensive rationale. This diversity seems to have its origin in the lack of a theoretical rationale that would guide researchers in the use of a methodology that is common to most of the studies. For example, many studies conceptualize beliefs as knowledge. However, the development of beliefs is not always based on cognitive and empirical knowledge, but rather on preconceptions, stereotypes, myths, and common sense. The theory of social representation [44] illustrates this argument very well. Alongside the absence of a theoretical rationale, the lack of a clear and common definition of “beliefs” also seems to explain the dispersion in the results, as well as in their interpretation. These theoretical and methodological limitations have implications for the validity of the evidence obtained in the study of the phenomenon. The fact that most studies had cross-sectional designs and that several used qualitative methods also limit the scope of the results, both in terms of internal and external validity, namely, compromising the possibility of generalization of those results. In this review, the lack of consensual information also exists regarding herbs/supplements. Some studies mention supplements, others mention dietary supplements, and others talk about herbal medicine. The lack of a consensual language, reflected in the use of several terms, makes it difficult for the reader, and for the potential users or sellers, to understand which substances are being documented in the literature. It is important to reinforce that healthcare professionals still have misconceptions about these products, and different opinions about their effectiveness and acceptability, which calls for the need of more scientific evidence on this topic, whether in terms of the substances’ effectiveness, indications or contraindications, and interactions with other products. 

Another difficulty in researching this topic has to do with the various countries where the reviewed studies were conducted. The first problem that this poses, and as previously mentioned, is related to the theory of social representations [44]. Cultural beliefs and the uses of herbs/supplements vary from country to country, resulting in the dispersion of the results. The legislation for these types of products in each country is also related to that culture’s social representations. For example, looking at the legislation of these products in various countries, a structured model is not followed across countries. Instead, countries define natural products in different ways and have different laws regulating their distribution, manufacturing, licensing, and safety concerns [111]. This not only reflects, and contributes to the development of different representations, but also affects how these products are labeled. All these aspects limit the capacity to organize all the data coherently. Furthermore, the deliberate consideration, in this study, of the perspectives of various target groups (health professionals, pharmacists and the general population) may require a careful reading of the results to avoid misleading interpretations.

As previously mentioned, most studies dealt with clinical samples, making it difficult to understand if the beliefs reported are a generalized social representation. Finally, it would be important to know more about the causal relationships between beliefs and practices associated with the consumption of herbs/supplements, but data on anchoring and objectification processes are not clear. 

## 5. Conclusions

In summary, it is imperative to improve the understanding and awareness regarding the utilization of herbs/supplements to enable healthcare professionals to prescribe and counsel patients on appropriate and optimal therapeutic interventions, and to empower users to access and share verified and reliable information pertaining to herb/supplement usage. Although health professionals and pharmacists seem to have more knowledge about HM, IPEDs, and HDIs than patients do, some studies still document their lack of knowledge, which requires in-depth attention. Further training in these topics is important and can be achieved, for example, through the inclusion of these contents in healthcare professionals’ formal training and through actions aimed at updating professionals who are already in the field. If technological solutions, such as the one proposed here, can contribute to address this problem, it is certain that it also brings new demands, namely the need for sharing information about the products purchased among different pharmacies. This requirement can become difficult to meet in view of the European General Data Protection Regulation. It might be important to inform the regulator/legislator about the possibility of creating exceptions, with the agreement of the patients, whenever health benefits are considered. Furthermore, although the technological solutions that are under development must be user-friendly technologies, it is important that professionals receive training in the use of the new means provided to support their practice. Healthcare professionals should also receive training in clinical communication, for informing and educating patients appropriately, allowing them to be involved in the decision process to make informed decisions.

Future research must incorporate a sound theoretical framework to facilitate the collection and analysis of relevant evidence. Additionally, researchers ought to conduct more experimental studies to establish the causal links between the beliefs, attitudes, knowledge and perceptions, and practices associated with herb/supplement use, as well as potential herb/supplement interactions. Moreover, informed supervision on the use of these products is crucial to ensure the safety of their users and should therefore be given due attention. 

Finally, recruiting participants from the general population, in addition to clinical populations, is important in the future. Nevertheless, for clinical populations, multidisciplinary teams must communicate among themselves to facilitate the monitoring of possible herb/supplement reactions and interactions, to generate new knowledge that can serve as a link between clinical practice and research. As illustrated by the awareness raised through already existing CDSS, the answers to these inquiries constitute a crucial component in the development of effective support strategies that can facilitate the timely detection of, and alerting for potentially hazardous reactions and interactions in any individual.

## Figures and Tables

**Table 1:** Correlational/comparative studies characterization.

1st Author (Year)	Domains/Dimensions Assessed in the Studies	Country	Design	Procedures for Data Collection	Sample (*n*)	Age (*M* ± *SD*) Min–Max	Gender
Makkaoui et al. (2021) [61]	Pharmacists’ attitudes, practices, resources and knowledge regarding drugs, food, and herb interactions.	Lebanon	Cross-sectional design	Questionnaires/surveys Individual interviews with a self-selected sample	Community pharmacists (89)	NR	NR
Al-Nadaf & Awadallah (2020) [62]	Knowledge and attitude about self-medicated use of herbal medicine and drug interactions.	Jordan	Cross-sectional design	Questionnaires/surveys	General population (926)	27.96 ± 14.1	F = 719 M = 207
Eltom et al. (2021) [63]	Knowledge, awareness, and attitudes of physicians toward the use of medicinal herbs.	Saudi Arabia	Cross-sectional design	Questionnaires/surveys	Physicians (117)	NR	F = 76 M = 38 NR = 3
Agrawal & Goel (2016) [64]	Use of herbal medicines; reasons for their use; source of information on herbal medicines; opinion on herbal medicines and their costs. Prevalence and perception of herbal drug usage in patients visiting the outpatient department (OPD) of a tertiary care hospital.	India	Cross-sectional design	Questionnaires/surveys	Patients (246)	18–30 31–40 41–50 51–60 >60	F = 119 M = 127
Koshak (2021) [65]	Which herbal medicines; attitudes and beliefs toward the use of herbal medicines in patients with allergies.	Saudi Arabia	Cross-sectional design	Questionnaires/surveys	Patients with allergic disease (111)	33 ± 18.0	F = 78 M = 33
Schiavo et al. (2017) [99]	Knowledge about medicinal plant herbal medicines.	Brazil	Cross-sectional and qualitative design	Interviews	Community health workers (13)	32.7 ± 8.19	F = 13
Soós et al. (2016) [66]	Attitudes and knowledge of workers in perioperative care toward Herbal Medicine.	Hungary	Cross-sectional design	Questionnaires/surveys	Anesthesiologists and surgeons (258)	39.9 ± 12.08	F = 107 M = 151
Lee et al. (2014) [67]	Prevalence of patients’ that use supplements; How oncologists communicated with patients regarding supplements. Oncologists’ knowledge, attitudes, and practice patterns regarding herbs and supplements use by their patients.	USA	Cross-sectional design	Questionnaires/surveys	Oncologists (392)	48.4 ± 9.8	F = 111 M = 277 NR = 4
Murtaza et al. (2012) [68]	Students’ knowledge about herbal medicines; knowledge about interactions.	Pakistan	Cross-sectional design	Questionnaires/surveys	Students (2830)	22 ± NR	F = 1265 M = 1515 NR = 50
Filho et al. (2021) [98]	Knowledge about medicinal plants used for the treatment of disease symptoms.	Brazil	Quantitative and qualitative design	Questionnaires/surveys	Students (60)	14–23	F = 36 M = 22 NR = 2
Chikafu et al. (2022) [95]	Awareness, beliefs, and health-seeking behavior about some diseases.	South Africa	Qualitative study	Focus group interview	General Public (76)	18–40>40	F = 41 M = 35
Santanello & Carr (2019) [69]	Perceptions and practices regarding herbal medicine.	USA	Cross-sectional design	Questionnaires/surveys	Community pharmacists (127)	20–64	NR
Atavwoda & Gabriel (2012) [70]	Pharmacists’ knowledge, attitude, and practices regarding herbal drug information services.	Nigeria	Cross-sectional design	Questionnaires/surveys	Pharmacists (273)	21–60	F = 130 M = 143
Pereira da Silva et al. (2018) [71]	Perception, knowledge, and attitudes of herbal medicine.	Portugal	Cross-sectional design	Questionnaires/surveys	Physicians (80)	51.9 ± 10.0	F = 57 M = 23
Thiab et al. (2021) [72]	Community perception of interactions (food/drink/medicine); knowledge of interactions.	Jordan	Cross-sectional design	Questionnaires/surveys	General public (789)	<18 18–25 26–40 40–65	F = 614 M = 175
Shraim et al. (2017) [73]	Knowledge, practices, and beliefs about complementary and alternative medicine.	Palestine	Cross-sectional design	Questionnaires/surveys	Community pharmacists (281)	20–29 30–39 40–49 50–59 >60	F = 132 M = 149
Dayer et al. (2016) [74]	How pharmacists maintain knowledge about the identification of the drug and/or herbal interactions and in the identification of adverse events.	USA	Cross-sectional design	Questionnaires/surveys	Pharmacists (246)	NR	NR
Al-Arifi et al. (2016) [75]	Knowledge about warfarin-herb interactions with drug and medicinal herbs.	Saudi Arabia	Cross-sectional design	Questionnaires/surveys	Physicians, pharmacists, and nurses (90)	25–35 36–45 46–55 >55	F = 37 M = 53
Nwose et al. (2017) [76]	Knowledge about the practice of using cassava in health.	Nigeria	Cross-sectional design	Questionnaires/surveys	General public (101)	<25 26–35 36–45 46–55 >55	F = 60 M = 41
Stanojević-Ristić et al. (2017) [77]	Attitude of the dietetic supplements used; perceptions of the effectiveness of dietary supplements; attitudes toward potential harmful effects and interaction with medicines; perception of the risk of adverse reactions to dietary supplements.	Serbia	Cross-sectional design	Questionnaires/surveys	Medical students (334)	23 ± 2.0	F= 188 M=146
Marx et al. (2016) [78]	Attitudes, beliefs, and behaviors regarding dietary supplements.	Australia	Cross-sectional design	Questionnaires/surveys	Dietitians (231)	<30 31–40 41–50 51–60 >61	NR
Stanojević-Ristić et al. (2022) [79]	Knowledge and behaviors regarding drug–dietary supplement and drug–herbal product interactions.	Serbia	Cross-sectional design	Questionnaires/surveys	General and specialist doctors, and nurses (346)	≤29 30–39 40–49 ≥50	F = 211 M =135
Oshikoya et al. (2013) [80]	Knowledge about the type of herbal medicines and their indications; knowledge about the use, contraindication and potential drug-herb interactions.	Nigeria	Cross-sectional design	Questionnaires/surveys	Pharmacists (103)	20–35 36–50 >65	F = 29 M = 74
Alaaeddine et al. (2014) [81]	Attitudes regarding the use of herbal medicines; knowledge about herbal medicines; general practices related to herbal medicine prescriptions.	Lebanon	Cross-sectional design	Questionnaires/surveys	Physicians (212)	49.18 ± 9.38	F = 107 M = 105
Jimam et al. (2017) [82]	Knowledge on herbal medicines; sources of information on herbal medicines; perceptions on herbal medicine.	Nigeria	Cross-sectional design	Questionnaires/surveys	Pharmacists (177)	34.0 ± NR	F = 48 M = 129
Tarn et al. (2020) [83]	Knowledge and prevalence of potential interactions with Apixaban and dietary supplements.	USA	Cross-sectional design	Questionnaires/surveys	Patients (791)	71 ± 11.8	F = 315 M = 472 Other = 4
Yan et al. (2021) [84]	Prevalence and preference of herbal products usage; perceptions of herbal products and awareness toward the drug-herb interactions.	Malaysia	Cross-sectional design	Questionnaires/surveys	University students (231)	22.0 ± NR	F = 111 M = 120
Sekhri & Kaur (2014) [85]	Attitude toward, and knowledge of multivitamin supplements, their consumption, and their effects.	General Public	Cross-sectional design	Questionnaires/surveys	General public (120)	F: 38.75 ± 12.87 M: 43.85 ± 15.44	F = 54 M = 66
Flower et al. (2015) [96]	Perceptions of herbal medicines; concerns about herbal medicines: knowledge, risk.	UK	Qualitative study	Interviews	General physicians (15)	44 ± NR 34-59	F = 7 M = 8
Younis (2019) [86]	Attitude, prevalence, and awareness toward herbal medicine products; their safety, information sources, the need to consult a physician prior to their use.	Jordan	Cross-sectional design	Questionnaires/surveys	Pharmacists (230)	35.4 ± 7.8	F = 142 M = 88
Santos et al. (2021) [97]	Behavior of consuming, concomitantly, boldo teas, cider grass, nuts skin, and lavender with traditional drugs.	Brazil	Qualitative study	Focus Group Interviews	Patients (12)	64–83	F = 9 M = 3
Jaber & Al-Zeidaneen (2021) [2]	Consumption of medicinal plants; the main indication of use for different postpartum health problems.	Jordan	Cross-sectional design	Interviews	Postpartum patients (300)	18–45	F = 300
Bhat et al. (2019) [87]	Knowledge and attitude of patients on the usage of herbal medicines.	India	Cross-sectional design	Questionnaires/surveys	Patients (322)	43.02 ± 14.33	F = 147 M = 175
Sridhar et al. (2017) [88]	Complementary and alternative medicine usage; perception, experience, and information-seeking behavior.	United Arab Emirates	Cross-sectional design	Questionnaires/surveys	General Public (403)	18–28 29–39 40–50 51–60 >61	F = 218 M = 185
Taing et al. (2017) [89]	Knowledge about popular herbal/nutrient weight-loss complementary medicines, their efficacy, potential side effects, and drug interactions.	Australia	Cross-sectional design	Questionnaires/surveys	Pharmacists (99)	33.5 ± 10.0	F = 61 M = 39
Alsayari et al. (2018) [90]	Knowledge, attitudes and practice regarding the indications, side effects, and contraindications of used herbal medicines.	Saudi Arabia	Cross-sectional design	Questionnaires/surveys	Pharmacists (233)	20–49	M = 233
Tank et al. (2021) [91]	Prevalence, motivation, and attitudes in the use of dietary supplements.	Germany	Cross-sectional design	Questionnaires/surveys and interviews	Cancer patients (1217)	67.6 ± 12.9	F = 624 M = 593
Albright et al. (2012) [92]	Reasons/motivations for taking dietary supplements versus prescription medications.	USA	Cross-sectional design	Questionnaires/surveys and focus group interviews	General public (396)	67.5 ± 7.4 52–88	F = 205 M = 191
el Khoury et al. (2016) [93]	Attitude and knowledge about medicinal drugs and dietary supplements.	Lebanon	Cross-sectional design	Questionnaires/surveys	Patients (726)	18–29 30–39 40–49 50–59 60–69 ≥70	F = 434 M = 292
Niveditha & Geetha (2020) [10]	Knowledge and awareness of natural anti-carcinogenic herbs and their uses.	General Public	Cross-sectional design	Questionnaires/surveys	General public (100)	NR	NR
Ceremuga et al. (2020) [7]	Knowledge about using herbal supplements; reasons for taking dietary supplements.	USA	Cross-sectional design	Questionnaires/surveys	Preoperative patients (2623)	18–24 25–29 30–39 40–49 50–59 60–69 70–79 ≥80	F = 1009 M = 1614
Mahnashi (2021) [30]	Knowledge, attitude, and practice about the use of herbal drugs.	Saudi Arabia	Cross-sectional design	Questionnaires/surveys	Pharmacists (62)	20–25 26–30 31–35 36–40 46–50	M = 62

F: Female; M: Male; NR: Not Reported.

**Table 2:** Experimental studies characterization.

1st Author (Year)	Domains/Dimensions Assessed in the Studies	Country	Design	Procedures for Data Collection	Sample (*n*)	Age (*M* ± *SD*) Min–Max	Gender
Diaz-Cruz & Bolten (2016) [94]	Knowledge and level of confidence regarding complementary alternative medicine.	USA	Experimental design	Formative and summative assessments ^1^	Pharmacy students (Phase 1–209) (Phase 2–38) (Phase 3–40) ^2^	23.8 ± NR	Phase 1 NR Phase 2 F = 30 M = 8 Phase 3 F = 32 M = 8
Miles Homer et al. (2019) [43]	Perceptions of dietary supplements.	USA	Experimental design	Intervention–Food and Drug Administration	Students 1st study (251) 2nd study (231)	1st study 23.5 ± NR 2nd study 22.8 ± NR	1st study F = 133 M = 118 2nd study F = 129 M = 102

Note: F: Female; NR: Not Reported; M: Male; ^1^ Formative assessment: two activities done in class and a reflection paper to evaluate material comprehension along the course; Summative assessment: midterm examination, an oral presentation, and a final practical examination; ^2^ The study was divided into 3 phases: Phase 1 consisted of questionnaires on the students’ preferences about the subject; Phase 2 consisted of the pre-course survey; Phase 3 was the post-course evaluation.

## Data Availability

Not applicable.

## Supplementary Materials

The following supporting information can be downloaded at: https://www.mdpi.com/article/10.3390/nu15102298/s1.
