# Supplementary material for: Knowledge and Beliefs about Herb/Supplement Consumption and Herb/Supplement–Drug Interactions among the General Population, including Healthcare Professionals and Pharmacists: A Systematic Review and Guidelines for a Smart Decision System
Source: Nutrients. 2023 May 13;15(10):2298. doi: 10.3390/nu15102298 (PMC10220613; doi:10.3390/nu15102298)
Supplement: Supplementary file 1 [file nutrients-15-02298-s001.zip › nutrients-2306313-supplementary.pdf]

Table S1. Knowledge, beliefs, attitudes, and perceptions about supplements, interactions and risks or adverse effects.

1st author (Year)	Main results (specific area)		
	**Consumption of supplements**	**Interactions between supplement and drug consumption**	**Consumption of supplements**
Makkaoui et al. (2021) [61]		Pharmacists search for information about interactions from the Internet, drug applications, or colleagues.<br>A flaw was found in the knowledge of the pharmacists about drug interactions with drugs, food, and herbs. The pharmacists had the right knowledge and attitude to report the interactions but needed the proper means to do so.<br>All respondents believe that it is important to check for drug–drug interactions, but the responses for drug–food interactions and drug–herb interactions were not unanimous, since many consider that food and herbs are safe because they are natural.<br>64.8% check interactions most of the time, but the rest believe that it is the physicians' job and not theirs.	

1st author (Year)	Main results (specific area)		
	Consumption of supplements	Interactions between supplement and drug consumption	Consumption of supplements
Al-Nadaf & Awadallah (2020) [62]	82% use self-prepared herbal preparation and use herbs as an alternative to medicines;<br>37% of the 82% use herbs as prophylaxis and 62% as treatment;<br>56% go for self-medication rather than visiting a physician;<br>63% say that physicians did not ask if they use any herbal medication;<br>50% not willing to tell their physician about their self-medication;<br>Knowledge about herbs gained from the family in the first place (38%), TV (21%) and friends (15%);<br>79% responders agree that herbs are safer and easier to be used when compared to physician-prescribed medicine;<br>Self-treat based on traditional beliefs (56.7%);<br>Herbs are used with the following pretexts: weight loss remedy, treatment for skin problems, anti-wrinkles remedy, cold and influenza, abdominal pain, headache;<br>Why people use herbs instead of standard therapy: herbal medicines are easier to be used, herbal medicines are cheaper to purchase.	Why people use herbs instead of standard therapy: have no side effects.	
Eltom et al. (2021) [63]	84% of doctors encounter patients who use herbal medicine;<br>Some patients (43%) liked to discuss herbal medicines with their doctors;<br>66% of doctors used herbal medicine for some ailment and 70% of them said it had beneficial effects;<br>70% of patients benefitted from the use of herbal medicines; Doctors were reluctant to discuss herbal drugs with their patients;<br>90% of the doctors had no opportunity to receive herbal medicine education.	60% of doctors knew that herbal medicines could have drug interactions with allopathic medicines.	68% of doctors knew that herbal medicines could have side effects.

1st author (Year)	Main results (specific area)		
	Consumption of supplements	Interactions between supplement and drug consumption	Consumption of supplements
Agrawal & Goel (2016) [64]	43.49% of patients reported using herbal drugs; 68.2% of patients use herbs on the advice of an alternate therapist; Among those who take herbs, 38.3% have revealed herbal intake to the treating physician; In non-herbal users, 71.3% had never given a thought to herbal drugs, as they lacked that knowledge; The main reasons for using herbs are: cure the illness; better life expectancy; safe; cure and safe; cure and better life expectancy; The main reasons for not using herbs are: never considered/lack of knowledge; satisfied from allopathic medicines or do not believe /seem unsafe.		
Koshak (2021) [65]	92% used herbal medicine concurrently with medications; 70% did not discuss their use of herbal medicine with their physicians; 50% used herbal medicine to increase disease control; 45% believed that herbal medicine is completely safe; Sources of information and advice: family (55%); Internet websites (31%), friends (19%), traditional healers (11%), textbooks (10%), physicians (6%), pharmacists (6%), television (5%), and social media (5%); Reasons to use herbal medicine: increased disease control (50%); safer than conventional drugs (42%) and due to limited benefit from conventional drugs (30%).	56% believed in herb-drug interactions.	

1st author (Year)	Main results (specific area)		
	Consumption of supplements	Interactions between supplement and drug consumption	Consumption of supplements
Schiavo et al. (2017) [99]	Lack of knowledge about herbal medicine;<br>More accessibility of plants and herbal medicines;<br>Know how to prepare preparation and storage plants;<br>The infusion was the most suitable preparation method;<br>The participants want to learn more.		The incorrect use of plants can cause health risks;<br>Medicinal plants and herbal medicines do not present a risk to health, because the "natural does not hurt."
Soós et al. (2016) [66]	Women were significantly more interested in alternative treatments than were men;<br>45.7 % do not know herbal medicine;<br>10% recommend herbal medicine to the patients;<br>29.1% consider it to be scientifically well-grounded;<br>7% claimed to use CAM;<br>86% cited scientific evidence as their knowledge source;<br>73.6 % consider it important that CAM methods should be taught in university settings;<br>66% supported the introduction of integrative medicine.		
Lee et al. (2014) [67]	Oncologists discussed herbs and supplements use with 41% of their patients;<br>26% of discussions were initiated by the oncologist;<br>64% indicated that they did not have enough knowledge to answer patients' questions about herbs and supplements;<br>59% had not received any education on the topic;<br>Estimated that > 40% of cancer patients use herbs and supplements;<br>Some had received some education that was gained through informal discussions (76%) or lectures (70%), conferences (38%), courses (14%), and other practices (31%).	93% were concerned about potential interactions between herbs and supplements and ongoing treatments;<br>Some participants were aware of some supplements-chemotherapy interactions.	

1st author (Year)	Main results (specific area)		
	Consumption of supplements	Interactions between supplement and drug consumption	Consumption of supplements
Murtaza et al. (2012) [68]	34.02% of pharmacy students responded that they are well-trained on herbal medicines;<br>95% of pharmacy students showed they have more knowledge than do the other students;<br>44% of students believed that phytotherapy is safe.	68.7% of the students believe that interactions may occur with herbal medicine interactions;<br>15.2% of students believe that there is no interaction with herbal medicines;<br>15.11% of students have no idea about interactions.	
Filho et al. (2021) [98]	Most students stated that they had previous knowledge related to medicinal plants;<br>Part of the students was not sure about their knowledge related to medicinal plants;<br>The use of plant extracts, and the consumption of home remedies increased during the COVID-19 pandemic;<br>Family, school, neighbors and social media are the most cited source of information.		
Chikafu et al. (2022) [95]	Men prefer self-care and herbal remedies, perhaps due to cultural and gender expectations;<br>Some participants believe that traditional healers and herbalists can cure some diseases but cannot heal others, like HIV, high blood pressure and diabetes;<br>Others believe that herbal remedies offer effective relief against hypertension;<br>The participants believe that Western medicine and traditional medicine should work together;<br>Financial constraints have been reported as a barrier to healthcare utilization.		

1st author (Year)	Main results (specific area)		
	Consumption of supplements	Interactions between supplement and drug consumption	Consumption of supplements
Santanello & Carr (2019) [69]	34% of community pharmacists were confident in their ability to effectively counsel patients about herbal medicines;<br>50% of pharmacists reported never or rarely asking patients about the use of herbal medicines;<br>80% reported never or rarely documenting the use of herbal medicines;<br>69% agreed that herbal medicines can provide beneficial health effects;<br>87% believed that it is important to be knowledgeable about herbal medicines;<br>79% believed that it is their responsibility to provide patients with information about herbal medicines;<br>49% agreed that they were not confident to provide adequate information about herbal medicine to patients;<br>69% of pharmacists reported never or rarely recommending herbal medicines;<br>63% reported occasionally or frequently recommending against the use of herbal medicines.	66% of pharmacists reported occasionally or frequently discussing herb-drug interactions when a patient purchases or inquires about an herbal medicine.<br>19% reported that they always discuss interactions between herbal medicines when patients are using herbal medicines.	64% of pharmacists reported occasionally or frequently discussing potential side effects when a patient purchases or inquires about an herbal medicine;<br>25% of pharmacists reported that they always discuss side effects.
Atavwoda & Gabriel (2012) [70]	46.33% reported having knowledge about herbal drugs while 64.0 % showed positive attitudes toward their use;<br>53.48% were aware of the laws and regulations controlling herbal drugs;<br>The most cited source of information on herbal medicine are the Internet and interaction with herbalist;<br>Herbal medicines are mostly used in fever, hypertension and infections/germs.		

1st author (Year)	Main results (specific area)		
	Consumption of supplements	Interactions between supplement and drug consumption	Consumption of supplements
Pereira da Silva et al. (2018) [71]	61.5% do not know about traditional herbal medicinal products legal recognition;<br>Lack of phytotherapy knowledge and training reported by 94% of physicians;<br>43% of the participants say they rarely or never ask patients about herbal medicine;<br>Gender difference in perceiving the effort required to obtain knowledge related to herbal medicine (women do the greatest effort);<br>Strong correlation between knowledge and willingness to use/prescribe;<br>Perception and positive attitude toward using/prescribing were positively correlated with the willingness to get training;<br>Training was considered to be very important.		
Thiab et al. (2021) [72]	70% used plants and herbs to improve their health;<br>66.4% of participants agreed that medicinal plants or herbs could treat diseases (mainly respiratory tract illnesses or immunity boosting);<br>45.6% believed that sometimes the treatment with herbs is better than it is with conventional drugs;<br>The most frequently cited information source was the Internet and physicians;<br>Positive perception toward herbs and their ability to treat diseases.	58.6% thought that medications could interact with drugs;<br>59.9% believed that children and the elderly are most likely to experience drug interactions with food, beverages or herbs.<br>The knowledge about interactions was poor.	

1st author (Year)	Main results (specific area)		
	Consumption of supplements	Interactions between supplement and drug consumption	Consumption of supplements
Shraim et al. (2017) [73]	Pharmacists frequently recommended CAM modalities;<br>96.5% agreed that alternative medicine need scientific testing before use;<br>Exercises (84.0%) and food supplements (82.6%) were the most frequently recommended modalities;<br>76% of participants agreed that the pharmacist must constantly inquire if the patient is using any type of CAM;<br>Participants showed an average score of 4.32 out of 8 when assessing their knowledge about CAM;<br>The study revealed that knowledge score increased as the pharmacists' experience decreased;<br>The most frequently cited information source was the Internet;<br>42% of participants held positive beliefs about CAM regarding the statement that results of CAM are mainly due to placebo effects.	12.5% of the participants gave a completely correct answer about drug-herb interactions.	49.1% disagreed that herbal drugs have less side effect than do conventional medicines.
Dayer et al. (2016) [74]	22% of participants reported confidence in their ability to manage an influx of oral antineoplastic agents;<br>Most pharmacists reported the main barrier to counseling to be a lack of training or knowledge;<br>The most frequently cited source of information about oral antineoplastic agents was package inserts, continuing education programs and drug information resources;<br>77% reported that their level of training or knowledge was the main barrier to counseling patients.		

1st author (Year)	Main results (specific area)		
	Consumption of supplements	Interactions between supplement and drug consumption	Consumption of supplements
Al-Arifi et al. (2016) [75]		66.7% of the responders identified the interaction between green tea and warfarin;<br>33 participants correctly classified warfarin interactions with cardamom;<br>Warfarin-drug interaction knowledge and herb-warfarin interaction knowledge had no significant difference between the health care professional groups;<br>Knowledge on warfarin-drug interactions and herb-warfarin interactions was poor and inadequate.	
Nwose et al. (2017) [76]	42% of the participants believed that cassava has medicinal value;<br>6% (among the 42%) believed that the plant is useful in treating diabetes;<br>24% of participants did not know the potential effects of cassava in diabetes management;<br>Participants with primary or no formal education believed on the medicinal value of cassava more than did the rest of participants with formal education.		

1st author (Year)	Main results (specific area)		
	Consumption of supplements	Interactions between supplement and drug consumption	Consumption of supplements
Stanojević-Ristić et al. (2017) [77]	53% of respondents used some form of dietary supplements;<br>The main reasons for using dietary supplements are to improve general health (49%), to strengthen immunity (39%), to improve nutrition (31%) and to enhance performance (24%);<br>The participants agreed that dietary supplements can be useful in the prevention of diseases, but were not sure if they can be useful for the treatment of diseases;<br>Attitudes regarding the safety of dietary supplements consumption showed a difference between the groups;<br>The participants agreed about the need of training and more education about dietary supplements;<br>In this study, physically active respondents were more likely to use dietary supplements;<br>Medical students educated in pharmacology showed a higher global perception of risk than did medical students not educated in pharmacology.		4% of respondents reported having experienced adverse reactions after taking dietary supplements (constipation, abdominal pain, sleep problems and nausea).
Marx et al. (2016) [78]	Dietitians are interested in dietary supplements (67%);<br>46% of participants believed that dietary supplements are important to improve health outcomes;<br>The most frequently cited source of information about dietary supplements are: Practice-based Evidence in Nutrition library (89%), guidelines (68%), academic journals (62%), and their colleagues (54%);<br>The results also indicate that Australian dietitians are tentative about integrating dietary supplements into their dietetic practice;<br>Strong interest in additional training in dietary supplements (79%).	The most frequently indicated barrier for use dietary supplements was concerns regarding potential interactions with other treatments (65%).	

1st author (Year)	Main results (specific area)		
	Consumption of supplements	Interactions between supplement and drug consumption	Consumption of supplements
Stanojević-Ristić et al. (2022) [79]		Sufficient knowledge about most major drug–dietary supplement and drug–herbal product interactions; Inadequate knowledge about most moderate interactions; Knowledge of the interactions was not associated with gender, type of practice, and postgraduate training; 28% of respondents reported that they often or always ask patients on drug therapy about their use of dietary supplements or herbal products; Nurses were more likely (64%) to often recommend the use of dietary supplements or herbal products to patients on drug therapy than were general practitioners (45%) and specialty doctors (33%); The knowledge of dietary supplements and herbal products was often not enough for them to manage drug–dietary supplement and drug–herbal product interactions; Healthcare professionals are aware that continuous education on this topic will be their necessity in future practice.	

1st author (Year)	Main results (specific area)		
	Consumption of supplements	Interactions between supplement and drug consumption	Consumption of supplements
Oshikoya et al. (2013) [80]	More male (53.3%) than female (44.7%) participants bought herbal medicines;<br>The pharmacists self-rated their knowledge of herbal medicines mostly as fair (39%) and good (42%);<br>The participants exhibited poor knowledge regarding the indications, contraindications and safety profiles of herbal medicine;<br>The most frequently cited source of information about dietary supplements are: medicines pack (56%) and the Internet (20%);<br>The information most frequently sought was, contraindications (75%) and adverse effects (70%).	The information most frequently sought was herb-drug interactions (85%),	
Alaaeddine et al. (2014) [81]	40% believed that herbal medicine was beneficial in patient care;<br>33.5% believed that herbal medicine was not safer than were conventional drugs;<br>43% of physicians believed that herbal medicine had the same activity as conventional drugs;<br>Poor knowledge about medicinal herbs;<br>80% acknowledged their need for more information at various levels;<br>45% routinely prescribed herbal medicine to their patients;<br>64 to 67% prescribers believed that herbal medicine has more benefits, faster results and fewer side effects than do conventional drugs;<br>58% thought that herbal medicine was less expensive;<br>76% believed that herbal medicine was easier to take than are conventional drugs;<br>Knowledge attitudes about the utilization of herbal medicine was not associated with age, gender or years of practice.	The physicians' knowledge about the possible mechanism of drug-herb interactions generally tended toward poor knowledge.	

1st author (Year)	Main results (specific area)		
	Consumption of supplements	Interactions between supplement and drug consumption	Consumption of supplements
Jimam et al. (2017) [82]	76.27% of participants believed that herbal products were more efficacious and safer (61.02%) than were orthodox medicines;<br>94.92% acknowledged the beneficial effects of incorporating herbal medicines into orthodox medicine practice;<br>72.88% lacked knowledge on herbal remedies;<br>The main source of information on herbs was school (56.50%);<br>67.80% of the respondents already used herbal therapy.	81.36% were not aware of any potential drug-herb interactions.	61.02% do not have any knowledge about side effects of herbal medicine.
Tarn et al. (2020) [83]	78.1% reported using dietary supplements daily or almost daily;<br>97.5% reported over-the-counter product use.	Dietary supplements with potentially serious interactions with apixaban were taken daily/most days by 20.2%;<br>93% spoke with physicians, and 46% spoke with pharmacists about whether it was okay to take an over-the-counter product with apixaban;<br>Less knowledge about over-the-counter products with potentially serious interactions was associated with greater over-the-counter product use.	

1st author (Year)	Main results (specific area)		
	Consumption of supplements	Interactions between supplement and drug consumption	Consumption of supplements
Yan et al. (2021) [84]	The awareness and knowledge about drug-herb interactions were low and inadequate among the participants;<br>The main source of information were family, friends, or relatives (86.6%) and the Internet (35.1%);<br>The main reason to use herbal products was to maintain their health condition (84.8%) and curing critical diseases (30.3%);<br>36.3% participants had taken herbal products together with conventional drugs;<br>Ethnicity and religion were factors associated with the herbal use perceptions.	Drug-herb interactions: herb leads addictive activity of drug (43.2%); herb affects drug elimination (51.1%); herb affects drug metabolism (47.9%); herb affects drug distribution (20.0%); herb affects drug absorption (47.4%); herb leads to bleeding (37.9%); herb affects drug efficacy (64.2%); general drug-herb interaction (59.5%).<br>Awareness and knowledge about drug-herb interactions were low and inadequate.	Awareness and knowledge about ADRs were low and inadequate.
Sekhri & Kaur (2014) [85]	68.33% of the participants were users of multivitamin supplements;<br>69.5% of the users consumed on the advice of their physicians;<br>The main reasons for the physician to have prescribed multivitamin supplements were: mouth ulcers, concurrent antibiotic prescription, arthritis, hypotension, weakness;<br>60% believed that multivitamin can be self-medicated;<br>70.96% of the users considered such supplements to be helpful;<br>Reasons quoted, namely, maintenance of general health (55%), to allay weakness or fatigue (20%), to improve the appetite (15%);<br>76% showed lack of knowledge about the natural sources of these vitamins.	The majority of the participants were unaware of the possible interactions of multivitamin supplements with other drugs.	The majority of the participants were unaware of the harmful effects of multivitamin supplements.

1st author (Year)	Main results (specific area)		
	Consumption of supplements	Interactions between supplement and drug consumption	Consumption of supplements
Flower et al. (2015) [96]	While some participants were open to the possibility of herbal treatment options, they required more research into effectiveness and safety, better regulation of herbal practitioners, and assurance about herbal quality control and potential herb–drug interactions;<br>Participants could recognize that herbal products can benefit the patients and have a "placebo" effect;<br>Concerns were expressed about the lack of quality assurance of herbal products, potential adulteration, and possible interactions between herbs and drugs and about the training and regulation of herbal practitioners.		
Younis (2019) [86]	41.7% used herbal medicine products;<br>58.3% preferred conventional medicine because it had better efficacy and better feedback than did herbal products;<br>The main sources of information were pharmacy college (47%), sales representatives (29%) and social media (19%);<br>The pathologies for which it was most frequently used were: gastrointestinal problems (25%), respiratory problems (9%) and obesity (9%).	40.4% do not have satisfactory knowledge about the adverse effects of herbal medicine products;<br>88.9% do not have enough information about the safety of these products and of herb-drug interactions.	
Santos et al. (2021) [97]	Reports of patients using complementary therapies in the treatment, such as the use of phytotherapy and of some teas;<br>With the traditional medication, the patients use boldo teas, cider grass, nuts skin and lavender to alleviate symptoms and/or assist in the treatment itself.	Patients do not consider that the herbs nullify or potentiate the effects of the drugs.	

1st author (Year)	Main results (specific area)		
	Consumption of supplements	Interactions between supplement and drug consumption	Consumption of supplements
Jaber & Al-Zeidaneen (2021) [2]	84% used one or more medicinal plants after delivery to control their postpartum health problems;<br>The main reasons to use herbal medicine were: decreasing post-delivery colic, flatulence and spasm (52.9%), treating maternal postpartum bleeding (41.7%), and lactation enhancement (41.0%);<br>9.0% of participants used herbals for weight control.		
Bhat et al. (2019) [87]	70.1 % were using herbal medicines and a combination of medicines;<br>34.07 % believed that herbal medicines are more efficacious, have fewer side effects and are safer than are conventional drugs;<br>The most frequently cited sources of information about herbal medicines were: friends (32.74 %) and drug advertisements (32.30 %);<br>64.6 % of the participants lacked knowledge on dose, duration of therapy, side effects and interactions of the drugs.		28.76 % of the participants could identify ADRs;<br>28.76 % of the participants could identify or felt ADRs.

1st author (Year)	Main results (specific area)		
	Consumption of supplements	Interactions between supplement and drug consumption	Consumption of supplements
Sridhar et al. (2017) [88]	The most commonly (52.1%) used complementary and alternative medicine among the participants was herbal medicine;<br>The pathologies in which it was most frequently used were diabetes and hypertension;<br>The most frequently cited source of information were friends/family members (67.7%) and TV/Internet/Newspaper/Radio/Magazines (36.5%);<br>64.5% do not discuss their complementary and alternative medicine usage with their doctor or other health-care professional;<br>22.6% of the survey respondents mentioned that they receive conventional medications;<br>52.9% of the survey respondents opted for complementary and alternative medicine to prevent illness;<br>51.6% of the survey respondents mentioned that complementary and alternative medicine usage was beneficial in improving their health condition;<br>53.6% believed that complementary and alternative medicines were safe and effective;<br>Age and ethnicity influenced the use of herbal medicine.		6% of respondents reported side effects or complications with the use of complementary and alternative medicine.
Taing et al. (2017) [89]	Pharmacists who self-reported having a good working knowledge were more likely to recommend weight-loss complementary medicines to their customers;<br>Participants have limited knowledge about weight-loss complementary medicines;<br>Participants do not recommend weight-loss complementary medicines frequently.	More than half of the participants were not sure/believed that there were no adverse effects/interactions associated with garcinia, green tea and chromium.	Pharmacists who reported a good working knowledge were able to identify one more of the 14 adverse effects than were those reporting knowing little/not remembering/never having heard of these weight-loss complementary medicines.

1st author (Year)	Main results (specific area)		
	Consumption of supplements	Interactions between supplement and drug consumption	Consumption of supplements
Alsayari et al. (2018) [90]	Pharmacists showed considerable knowledge of the indications of herbal products;<br>60.9% believed that herbal medications are efficient;<br>45.1% believed that herbal products should be sold only in a pharmacy;<br>44.2% believed that the use of herbal medicine should not be limited to patients who have failed traditional prescription therapy;<br>79.4% “strongly agreed” or “agreed” that it is a pharmacist's responsibility to provide information about herbal medications;<br>47.2% “agreed” that continuing education on herbal medications should be mandatory;<br>42.1% “sometimes” use herbal drugs for self-treatment;<br>The pharmacists of this study exhibited good practices toward herbal medicine.	70% of the participants could recognize possible side effects, contraindications, and drug-herb interactions.	
Tank et al. (2021) [91]	47.2% of patients reported using dietary supplement;<br>Women (56.3%), patients with an academic degree (56.0%) and non-smokers (84.8%) were more inclined to use a dietary supplement;<br>Women (84.8%) and patients younger than 65 years (84.4%) sought dietary advice more often;<br>The main reasons to use dietary supplement were: to support the immune system (26.4%), prevent nutritional deficiencies (18.8%), improve quality of life (15.7%), defeat cancer (11.8%);<br>The main sources of information were: print media, oncologist and friends or family.		

1st author (Year)	Main results (specific area)		
	Consumption of supplements	Interactions between supplement and drug consumption	Consumption of supplements
Albright et al. (2012) [92]	The most frequently mentioned reasons for taking supplements were maintaining a healthy life (64%), recommendation by healthcare professionals (51%), and prevention of disease/medical problems (50%);<br>High proportions of foreign-born Latinos and Japanese-Americans reported that dietary supplements were as important as were prescription medications;<br>76% of participants reported that their dietary supplements were as important as were prescription medications.		
el Khoury et al. (2016) [93]	35% reported to be currently consuming at least one herbal product/dietary supplement;<br>64% of those who reported taking herbal products/dietary supplements purchased them in pharmacies, and 32% at nutrition/supplement stores;<br>The main sources of information were: family member, friend or relative (39.5), pharmacist (21%) and physician's recommendation (19.7%);<br>15% of male and 29.2% of female participants believed that minerals and vitamins are not dangerous;<br>34.9 % of participants believed that insurance should cover supplements;<br>85.9 % expressed an interest in getting more information.	20% had documented drug–supplement interaction incidents.<br>13% of consumers were identified as having at least one supplement–supplement interaction event.	
Niveditha & Geetha (2020) [10]	About 69% of people think that cancer can be controlled by naturally available herbs;<br>85% of people are aware that grape seed extract possesses anticancer properties;<br>The general public have moderate knowledge and awareness of natural anti-carcinogenic herbs and their uses.		

1st author (Year)	Main results (specific area)		
	Consumption of supplements	Interactions between supplement and drug consumption	Consumption of supplements
Ceremuga et al. (2020) [7]	32.3% of respondents reported at least 1 dietary supplement use;<br>75.3% reported taking the dietary supplements daily;<br>66.4% believed that dietary supplements help the condition for which it is taken;<br>58.8% were very or extremely confident that the dietary supplements function as claimed;<br>84.4% were very or extremely confident that the dietary supplements are safe;<br>The most frequently cited reasons for using the dietary supplements were deficiencies (14%) and overall health (12.4%);<br>Dietary supplements research is needed, and patient education should be incorporated routinely during preoperative encounters.	Lack of knowledge regarding the potential interactions;<br>97.1% lacked knowledge regarding any potential medication interactions.	Lack of knowledge regarding side effects;<br>89.7% of patients taking dietary supplements were not aware of any potential side effects.
Mahnashi (2021) [30]	63.6% of the respondents "disagreed" or "strongly disagreed" that herbal medications are not safe and effective and that they have similar activity as do synthetic drugs;<br>53.2% "disagreed" or "strongly disagreed" that herbal medicine should be used only by patients who do not benefit from conventional prescription drugs;<br>51.6% "sometimes" used herbal medications for self-treatment;<br>62.9% believed that people trusted using herbal medicines;<br>77.4% also believed that people will continue using herbal medicines;<br>59.7% of the respondents believed that people are not educated enough about herbal medicine;<br>Pharmacists showed good knowledge, a positive attitude, and efficient practice toward herbal medicines;<br>There is a demand for further training, continuing medical education.	63.54% recognized correctly the possible adverse effects, contraindications, and drug–herb interactions related to the selected herbs.	

Table S2. Knowledge, beliefs, attitudes, and perceptions about supplements, interactions and risks or adverse effects of the included experimental studies

1st author (Year)	Main results (specific area)		
	**Consumption of supplements**	**Interactions between supplement and drug consumption**	**Consumption of supplements**
Diaz-Cruz & Bolten (2016) [94]	The knowledge about dietary supplements improved after the course, participants were more confident to discuss this topic with colleagues and patients.	The knowledge about dietary supplement/prescription drug interactions improved after the course	The knowledge about reporting and documentation of adverse effects due to dietary supplement use improved after the course
Miles Homer et al. (2019) [43]	Multi-ingredient supplements possess more severe adverse side effects when compared with single-ingredient supplements;<br>A disclaimer intervention that draws attention to the lack of government regulation and testing of supplements was ineffective at influencing perceived side effect severity;<br>Lay theories impact dietary supplement decision making.		
